# Valorization of products from grounded-coffee beans

**DOI:** 10.1038/s41598-021-99938-x

**Published:** 2021-10-14

**Authors:** Shawn Gouws, Michael Muller

**Affiliations:** 1 Department of Chemistry, Nelson Mandela University, Gqeberha, 6031 South Africa; 2 Nelson Mandela University, InnoVenton, Gqeberha, 6031 South Africa

**Keywords:** Chemistry, Materials science

## Abstract

The valorisation of ground coffee beans is discussed in two parts; the first research question relates to the extraction of cold brew from ground coffee beans to provide a healthy cold beverage. Two parameters were investigated: temperature, and the ratio of ground coffee beans to water. This work suggests that cold brew coffee can be extracted between 15 and 20 °C over 2 to 4 h instead of 24 h as outlined in typical cold brew extraction processes. The coffee aroma was the response variable. Part of this investigation was to develop a downstream product from the waste spent grounded coffee bean. This part of the study investigates the production of firelighters from spent ground coffee beans to reduce the impact of dumping significant quantities of spent coffee grounds from coffee houses, restaurants, and baristas on landfill sites, which can lead to environmental problems such as polluting water systems, killing wildlife and disturbing ecosystems. The study used spent ground coffee beans in products such as firelighters to test their efficacy. This application has shown promising results, with the firelighters showing longer burning times for the ignition of log fires while also emitting a gentle, pleasant coffee aroma.

## Introduction

For many people, drinking coffee is part of a daily routine to start the day^1^. Coffee is a comforting beverage that influences mood and satisfies thirst.

Coffee consumption in South Africa has increased steadily from 29 760 tonnes in 2012/13 to 55 000 tonnes in 2016/17^2^. A survey by Masterton’s coffee showed that consumers want greater varieties of flavour and different experiences from their coffee drinking^3^. Cold-brewed coffee (CBC) could be a pleasant alternative as a cold beverage for summer months launching a new experience for coffee drinkers while still allowing them to enjoy the kick caffeine brings in a hot-brewed coffee drink. Cold-brewed coffee is not iced coffee (which is hot-brewed coffee over ice); CBC is prepared at room temperature over a 12 to 18-h period compared to quick, traditional hot brewing methods^4–6^.

Brewing coffee is an extraction process that depends on several factors, such as the ratio of water to ground coffee beans (GCB)^7^, the temperature of the water, the diameter of the ground coffee particles (coarse, medium or powder), and the brewing time. The temperature significantly influences the aqueous solubility of the compounds, and the literature records a significant difference in composition between hot brewing and cold brewing^8^. Much literature has been published detailing the chemistry of hot water brewing, including quantification of the caffeine concentration as a function of the hot water brewing method^9,10^. During the hot brew extraction much more of the caffeine and caffeinic acids are extracted from the coffee bean giving hot brew coffee that rich coffee aroma and bitter taste.

Coffee grounds contain a mixture of volatile^11^ and non-volatile components, such as various oils, acids, and other aromatic molecules^5^. Collectively, these compounds are referred to as "coffee-soluble” and contribute significantly to the flavour of the coffee. Factors such as temperature affect the solubility and volatility of these compounds^5^. Solubility describes the number of solids dissolved out of the GCB into the water, where volatility refers to their ability to evaporate into the air. It is the increased volatility at higher temperatures that releases the aromatics more easily, giving rise to the enticing aroma of freshly brewed coffee^5^. What cold brewed coffee lacks in temperature, it makes up for in brewing time^4^. Increasing the time of extraction from a few minutes to hours aims to maximize the extraction of the solutes from the GCBs. Other factors, such as oxidation and degradation, can still occur in cold brewed extraction methods, but this happens much more slowly than in the hot brewed method. A decrease in bitterness and acidity are also reported in cold brewing^12^, especially if the CBC is kept cold; in consequence CBC extracts might take on a much sweeter, floral characteristic. Cold brewed coffee has been reported to possess a different flavour profile, with a typical characterization of aroma, acidity, aftertaste, body, flavour and sweetness that differs from other coffee extraction processes^13^.

The large quantities of coffee consumed worldwide everyday result in immense quantities of spent coffee grounds. The environmental impact of dumping spent ground coffee beans (SCGB) is enormous; hence other the need to find ways to use the SCGB to produce other commercial products^14^.

Part of this investigation is to develop downstream products that will make use of the spent coffee beans as a more valuable commodity product that can be used by the end user. The second section of the paper describes the production of firelighters from SCGB. Normally, these residues would be dumped as fertilizer to condition soil, or used as a green alternative in construction materials^15^, but other higher value products, such as firelighters or burning bricks for heat generation, are possibilities. The grounds could be used as an exfoliant for cosmetic products, in biofuel^16^, or as a source of extracted antioxidants^17,18^.

In this research, the authors focused on firelighters as a possible alternative product for the SGCB.

The objectives of this study were to the development of a range of coffee-based beverages using a cold-brew process, develop quality assurance protocols for the cold-brew extract, evaluate methods to improve the shelf-life of the final beverage, and to beneficiate the coffee grounds resulting from the cold-brew process. The following 3 core objectives will be discuss in this paper; (1) to determine the optimum extraction time and temperature for a specific coffee bean from Rwanda and so extend the consumption of coffee into the hot summer months, (2) to utilize sensational criteria to determine the best dilution of CBC extract with water or as a freeze-dried product to study the best coffee aroma dilution, (3) to utilize the SGCB to manufacture firelighter as an alternative to fossil fuel firelighters.

**Figure 1:**
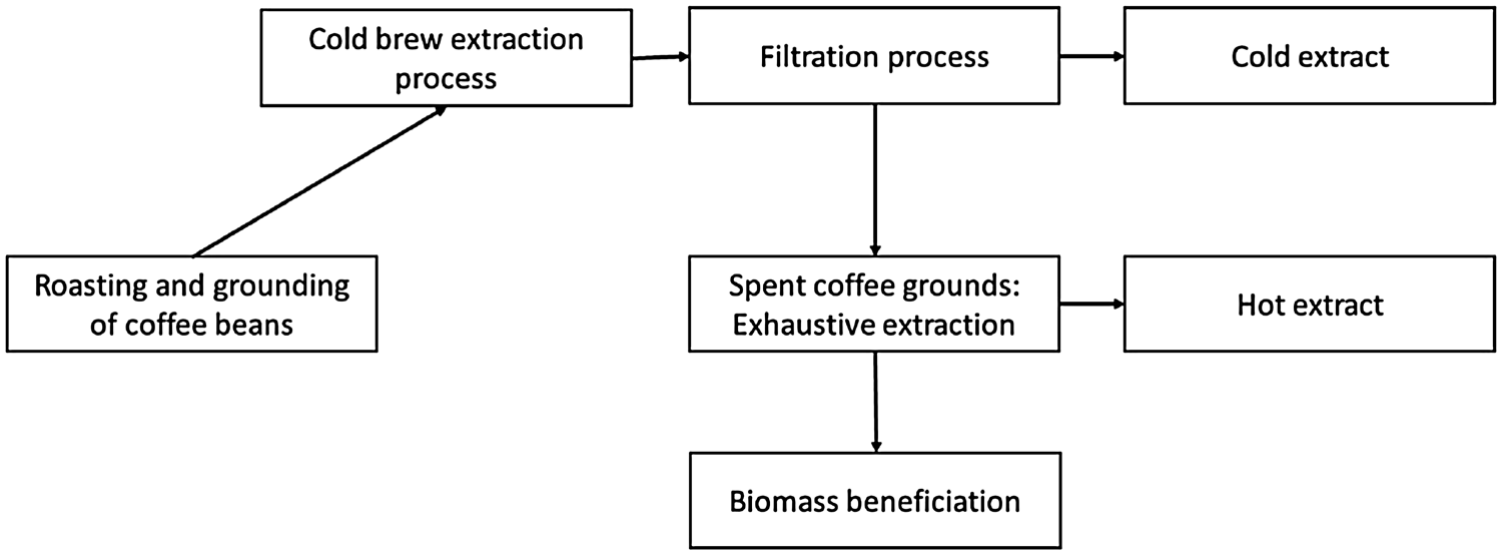
Cold brew extraction process*.*

The proposed project is illustrated diagrammatically below (Fig. 1):

## Materials and methods

### Raw materials

Roasted coffee beans (medium) of Rwanda obtained from a local vendor, Masterton’s Coffee House, Gqeberha (Port Elizabeth), South Africa.

### Sample preparation

Coffee beans were coarsely ground with a commercial coffee grinder.

## Cold coffee extraction

The goal of this experiment was to determine the optimum temperature at which CBC should be brewed to yield the most product between 15 and 20 °C. Extractions of the coarse GCB were carried out between 2 and 4 h at ratio of coffee to water of 1/5, 1/7 and 1/12. The effect of these factors was monitored by measuring pH and total dissolved solids (TDS).

### Total dissolved solids

Total dissolved solids were measured according to previously described methods^19^, and the total weight of water mass divided by the GCB weight used for each extraction.

### Pilot-scale demonstration of the CBC extract

A small-scale pilot demonstration was performed at a sister Technology Station—Agrifoods at Cape Town University of Technology. A reactor was charged with coarse ground Rwandan coffee beans (720 g) to which water (5 L) was added. The mixture was agitated slowing at 150 rpm for 12 h at 20 °C, filtered to remove the spent coffee beans, after which the CBC extract was freeze dried. Freeze drying the product will stabilize the product giving it a longer shelf-life.

### 3D—Surface plots

The CBC extractions and the freeze-dried granular products were prepared, and a in house panel consisting of the authors to rate the coffee, according to aroma, acidity, aftertaste, balance, body, and flavour. The tasting method as described in literature^20^ was utilized. From these results, 3D surface plots were constructed to examine the relationship between three response variables by viewing a three-dimensional surface of the predicted response.

## Spent ground coffee bean preparation

Spent ground coffee beans (SGCB) were mixed with wax and molasses and placed in a mould to set. Once set, a weight sample was set alight, the burning time was measured and compared to a commercial firelighter of the same weight.

## Results

### Part 1: CBC extraction

During these experiments, it was noted that the extraction proceeds more rapidly than the 18-h suggested in the literature.8 All the different ratios reached their end point in just under four hours. This means time can be saved and more than one batch can be prepared in a single day^4^.

To verify the results, two extractions of two hours and four hours each were performed. Table 1 summarizes the results. A 100 ml sample was taken from each of these extractions and freeze-dried. The yield percentage was then calculated and compared to the TDS readings and to previously reported 18-h extractions.

**Table 1:** Results of cold brew extractions after 2 and 4 h.

	2 h	4 h
Total extraction volume (ml)	300	300
Amount of coffee grounds (grams)	42,87	42,95
Freeze dry sample volume (ml)	100	100
Freeze dry mass (grams)	2,26	2,67
Calculated mass for overall extraction	6,78	8,01
Yield %	15,82	18,65

**Figure 2:**
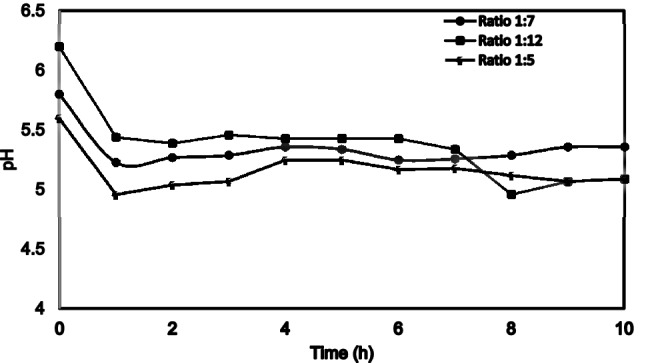
Effect of pH on the CBC extract.

Figure 2 shows the combined effect of pH results for the three GCB to water ratios at 1/5, 1/7, and 1/12 (volume:mass) mixture ratios respectively. There was no significant difference observed between the three ratios and the coffee extracts. The pH observed was between 4.5 and 5.5 which indicates the caffeic acids present in the coffee extract^21^.

**Figure 3:**
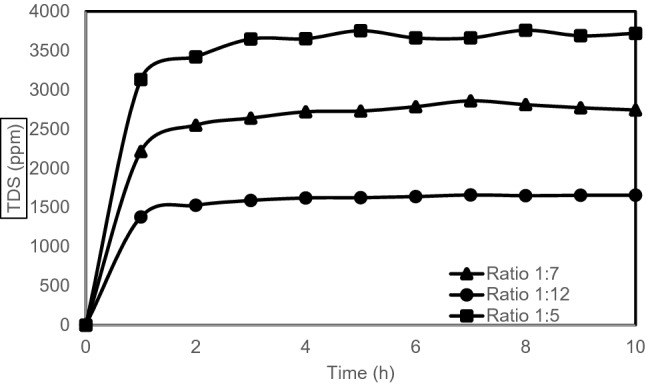
TDS effect over time.

The TDS results (Fig. 3) show that the ratio between GCB and water became constant after 1 to 1.5 h for all three GCB to water ratios, regardless of the concentration of the GCB in the cold water. As with the pH readings, after 1 to 1.5 h the extraction was complete.

The CBC extraction samples were subjected to various criteria to grade the CBC extract. A chart was constructed with the following criteria: aroma, flavour, acidity, body, aftertaste, and balance.

The aroma was chosen because of the effect aroma has on taste. Coffee aroma has several attributes that could enhance the coffee favour, other than the mouthfeel and the sweet, salt, bitter, and sour taste attributes that are perceived by the tongue^22^. The CBC needs to have a good, full-body taste and not be flat or bland. Aroma is detected by two different mechanisms: it is sensed nasally via smelling the coffee, or by retro nasal perception which occurs when the coffee is either present in the mouth or has been swallowed and aromatic volatile compounds drift upward into the nasal passage^23^.

Acidity has nothing to do with the amount of acid present or the pH; it describes a coffee taste that refers to the fruity, tangy, wine-like flavour that characterizes many Arabica coffees^24^. The acidity becomes more prominent in longer roasting times of GCB.

“Body” describes the texture of the cold brew coffee; the full-bodied coffee taste refers to the strong, soft, and enjoyable feel of the coffee in the mouth. A coffee's body (light, medium, or full) refers to the depth of the aroma on the palate caused by the amount of dissolved and suspended solids and oils extracted from the coffee grounds. This may range in thickness from a thin, watery texture to a thick, creamy texture^25^.

The aftertaste refers to the taste of brewed coffee vapours released after swallowing. Also called "finish", aftertastes can be burnt, chocolatey, spicy, etc.

A balanced coffee may be complex but does not have any overwhelming flavour or aroma characteristics.

Most mixes were made up in a ratio of one teaspoon of coffee to a cup of water. Table 2 summarizes the CBC extract results which revealed that the most favoured test sample seemed to be the roasted coffee bean extract that was extracted in a 1:7 (v/v) CBC: water mixture ratio. Most of the tasters liked the 1:7 (v/v) freeze-dried sample. Sample 4 consisted of the fresh extract CBC in a 1: 2 (v/v) dilutions with cold water. This small population of tasters indicated that the ground roasted coffee bean, coarse particle size in a 1:7 (v/v) ratio to the water extract medium gave the best cold brew extract coffee according to the taste parameters.

**Table 2:** Summary of cold brew extraction test results.

Run	Sample name	Aroma	Flavour	Acidity	Body	Aftertaste	Balance
s1	1:14 Diluted 1:1	81	69	**81**	50	**75**	**69**
s2	1:7 Diluted 1:3	75	56	64	39	67	61
s3	1:14	81	56	58	58	50	56
s4	1:7 Diluted 1:2	**89**	56	58	47	58	64
s5	1:7 Diluted 1:1	86	67	58	58	53	56
s6	1:14 Freeze dried, 2,6 g/120 ml	78	67	58	56	61	64
s7	1:7 freeze-dried, 1,3 g/90 ml	67	**72**	67	58	75	64
s8	1:7 freeze-dried 2,6 g/90 ml	75	58	58	**61**	42	56
s9	1:14 After 1 week	53	69	69	53	67	61
s10	1:7 Diluted 1:1 After 1 week	58	64	61	58	64	64

**Figure 4:**
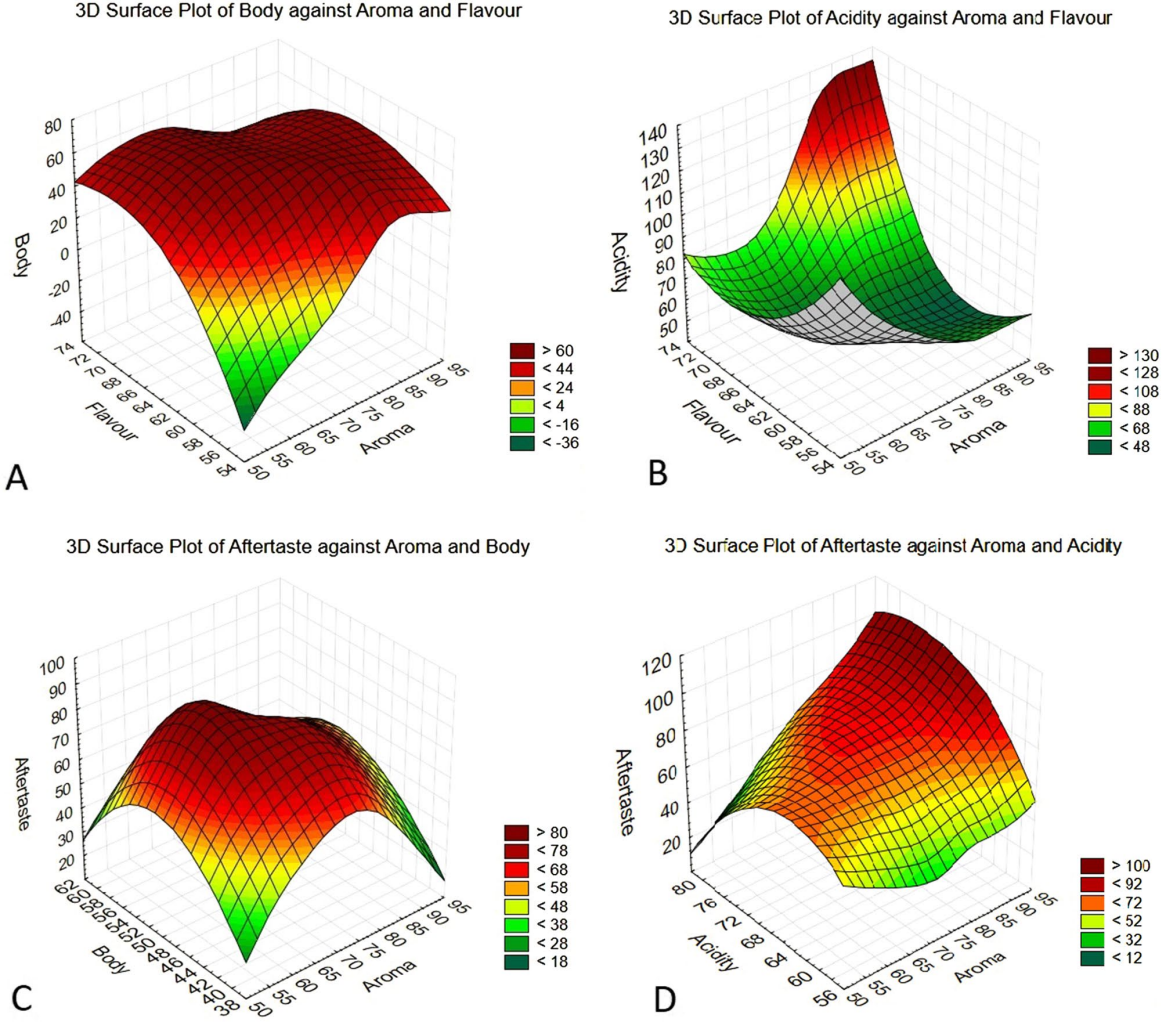
3D surface model plots to examine the relationship between response variables. (**A**) comparing aroma, body, and flavour, (**B**) Comparing aroma, flavour, and acidity, (**C**) comparing aroma, body, and aftertaste, and (**D**) comparing aroma, acidity, and aftertaste. The key is a percentage value.

The 3D surface model plots showed that when the sample has a good body, the aroma and flavour improve when compared to diluted samples or weaker ratios of extract coffee to water. Figure 4a shows the 3D surface plot for aroma, flavour, and body.

From the 3D surface model plot (Fig. 4b) which shows aroma, flavour, and acidity, it is clear that almost all the tasters like the acidity of the cold brew extract as the aroma becomes fuller in the body. Figure 4c is the 3D surface model plot for the comparison between aroma, body, and aftertaste; the comparison of these three parameters shows the tasters agreed that the CBC extract have a good aftertaste and coffee aroma.

Figure 4d shows the comparison of aroma, acidity and aftertaste. As the acidity increases, so does the aroma, and there were some improvements in the aftertaste of the coffee that was extracted at a 1:7 ratio.

#### Freeze drying

These results demonstrate that a 4-h brew yields 18% freeze-dried solid mass, compared to the 16% during a 2-h cold brew extraction. It was observed that the optimum cold brew extraction is in the region of about 4 h compared to the 12–18 h suggested by other sources^8^.

#### Temperature determination

By analysing the results of the experiments at 10, 15 and 20 °C we can determine how the temperature affects the cold brewing of coffee. By keeping all factors constant except the temperature changes, we were able to compare the yields of these experiments. From the results, we can conclude that the temperature at which coffee is cold brewed, does not affect the quality of the product yield. This means we can save energy costs by not needing to cool the setup to 10 or 15 °C, but rather brewing at 20 °C. The cold extraction technique was compared to hot extraction at 95 °C. In each study, it can be observed that a smaller amount of TDS was extracted using hot water than extracts from the cold-water extraction. The amount TDS extracted ranged between 1021 and 1050 ppm compared to over 2000 ppm for the cold brew extraction. The pH remained the same at 4.88 for all the extractions, indicating that the caffeinic acid been extracted from the ground coffee beans. The amount of coffee extracted from the GCB during the cold brew extraction process gave a yield of 19% compared to the hot extraction method that resulted in only a 6% freeze-dried solid mass percentage.

#### Cold brew extraction

Table 2 summarized seven CBC dilutions and three freeze-dried CBC granular to water ratio samples that were prepared for sensational analysis to identify the best coffee aroma characterization. The characterization and interactions were to taste for acidity, aftertaste, aroma, balance, body, and flavour. The purpose of this test was to establish which product would be best to take to market and in what format. Most commercial coffee sellers prepare the CBC in advance and store it in a keg, in a cool place in order to achieve a good quality brew extract; they then filter the CBC before serving. The authors suggest that, in order to achieve a longer shelf life, freeze-drying the CBC extract would remove caffeinic compounds from the coffee and provide a better, healthier option, rich in caffeine but without the caffeinic aromatics.

Table 2 indicates that the CBC extract, in most cases, appears to be flat in comparison to hot brew (traditional way) coffee and scores a 61% for the 1:7 freeze dried product (2.6 g/90 mL). The best overall ratio was the CBC extract dilution of 1:14, further diluted 1:1 with water. This was scored overall as 71%, followed by Sample 7, the 1:7 freeze-dried product, with a score of 67%. The panel enjoyed the overall taste of cold brew coffee and stated that although the body of the CBC is flatter than hot brew coffee extract, the CBC taste is more balanced and has a sweeter taste. Cold brew coffee extract also has a soft coffee aroma and aftertaste.

### Part 2: uses of spent ground coffee beans (SGCB)

Since the aim of this research was to utilize the entire GCB, it was important to study a case where they could be used as firelighters. There are other uses for spent beans, such as a fertilizer for soil conditioning, or a meat rub before BBQ, and so on. However, some entrepreneurs suggested investigating firelighters. The method was discussed under “Materials and Methods” and the results are given below. Utilization of waste material such as the spent GCB can be used for fertilizer or be used to make firelighters. The following discussion outlines the results obtained from producing firelighters from SGCB.

The length of burning time of these firelighters was compared to commercial firelighters; on average they burn for about 9.45 min compared to commercial firelighters which burn for 10.45 min per 20 g.

#### Firelighters

The firelighters were constructed from three components: SGCB, molasses, and wax. The SGCB provided the filler, the wax the fuel, and the molasses the binder. Two ratios were used for the firelighters 2:1 and 1:1 SGCB to wax. With each ratio, molasses was added to one sample as a binder to see if it changed the way the firelighter burned, so in total, 2 samples of each ratio were prepared. The sample size of the firelighter is relatively big and will be made smaller for commercial purposes. The size of the test firelighters manufactured from the SGCB and the commercial ones was roughly between 20 and 25 g.

#### Results

The 20 g, 1:1 SGCB:wax ratio firelighters stayed alight for 9.45 min compared to the 20 g, 2:1 SGCB:wax ratio which burned for 16.05 min. In comparison, a commercial product stayed alight for about 10.45 min.

**Figure 5:**
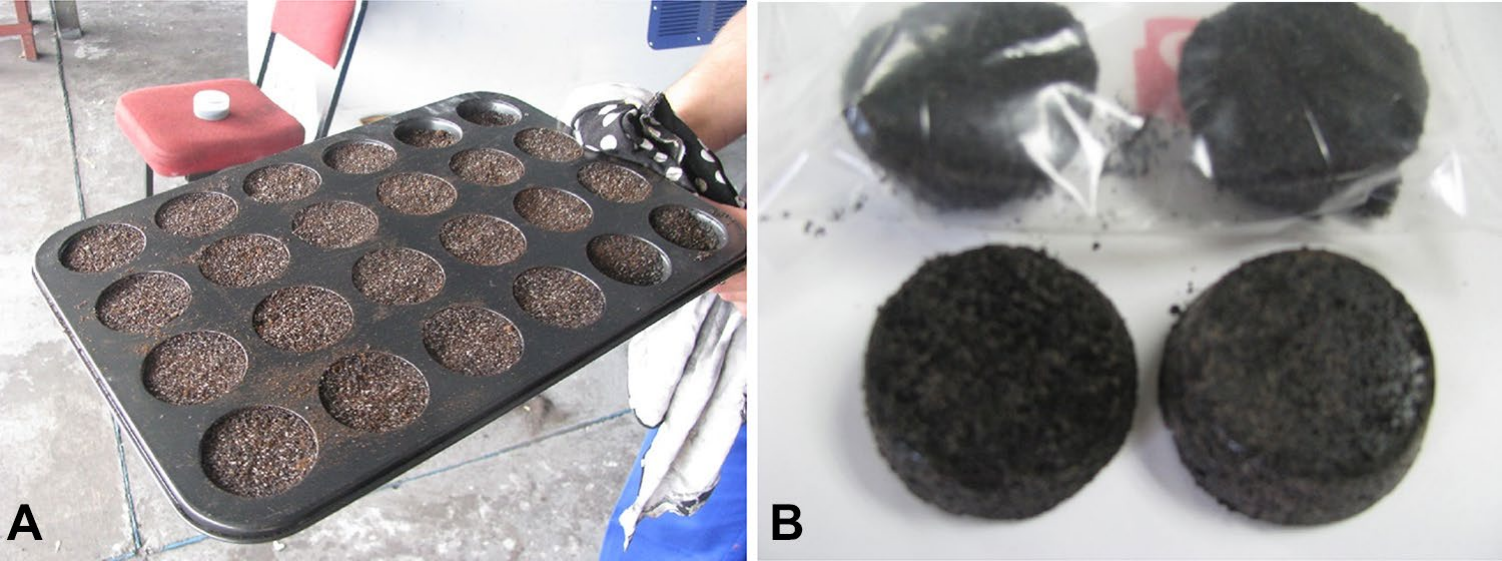
(**A**) Firelighters after casting. (**B**) The finished product.

Table 3 summarizes the results of the burn test of the commercial firelighters against the laboratory prepared coffee firelighters (Fig. 5). Commercial firelighters only achieved an average burn time of 10 min and 45 s. The 2:1 (m/m) coffee to wax firelighters performed the best and burned for 16 min. The addition of molasses as a binder seems to have increased the burn time by 45 s, but by observation, a binder is not necessary for this ratio because, without the molasses, the moulds kept their shape throughout the burn test.

**Table 3:** Burning rates for each coffee firelighter and the commercial one.

Coffee Firelighter	
Ratio Coffee: Wax	Average burn time in minutes
1:1	9:45
1:1 + Molasses	10:05
2:1	16:05
2:1 + Molasses	16:50
Commercial fire lighters	10:45

The ratio of 1:1 (m/m) did not perform as well as the 2:1 (m/m) ratio, achieving a burn time of only 9 min 45 s. Even with the addition of molasses as a binder, the burn time reached only 10 min. Observation confirmed that the 1:1 ratio contained too much wax, making the binding less firm than that of the 2:1 ratio; thus, as it started to burn, it fell apart very early on, so reducing the burn time.

The 2:1 ratio seems to be the best firelighter medium, surpassing even the commercial firelighter (Fig. 5).

## Conclusion

The research shows that the cold brew coffee could be extracted in four hours with a GCB to water ratio of about 1:7 (m/v) mixture. This ratio produced the best aroma and acidity, giving a fuller bodied texture. Particle size was shown to have no significant effect on the extraction process.

Future recommendations would be to continue with the 1:7 CBC extract in the water at room temperature but on a slightly bigger scale of about 5–10 L with and without agitation, under air and nitrogen atmosphere. This approach could provide good evidence about the feasibility of the project. Further studies could be done on freeze-drying the CBC extract or investigating suitable packaging materials by making use of sachets under dry conditions or using concentrates.

The firelighters proved to be a possible alternative use of the SGCB rather than merely disposing of it on a compost heap or using it as fertilizer. Making firelighters adds value to a spent product and could be used to start an entrepreneurial business.

## Abbreviations

CBC: Cold brew coffee
GCB: Ground coffee beans
SGCB: Spent ground coffee beans
TDS: Total dissolved solids
v/v: Volume to volume ratio
